# An investigation into inflection-point instability in the entrance region of a pulsating pipe flow

**DOI:** 10.1098/rspa.2016.0590

**Published:** 2017-01

**Authors:** J. J. Miau, R. H. Wang, T. W. Jian, Y. T. Hsu

**Affiliations:** Department of Aeronautics and Astronautics, National Cheng Kung University, Tainan 70101, Taiwan, Republic of China

**Keywords:** inflection-point instability, pulsating pipe flow, unsteady boundary layer, hot-wire measurement

## Abstract

This paper investigates the inflection-point instability that governs the flow disturbance initiated in the entrance region of a pulsating pipe flow. Under such a flow condition, the flow instability grows within a certain phase region in a pulsating cycle, during which the inflection point in the unsteady mean flow lifts away from the viscous effect-dominated region known as the Stokes layer. The characteristic frequency of the instability is found to be in agreement with that predicted by the mixing-layer model. In comparison with those cases not falling in this category, it is further verified that the flow phenomenon will take place only if the inflection point lifts away sufficiently from the Stokes layer.

## 1. Introduction

The well-known Reynolds experiment [1] is concerned with the laminar–turbulent transition phenomenon in a pipe flow for which the mean flow is steady. In this study, our main interest is focused on the laminar–turbulent transition process in a pulsating pipe flow. This situation is relevant to many areas of application, including fluid flows in bio-systems and the transportation of fluid in industrial pipe lines.

An important feature of the laminar–turbulent transition process in a pulsating pipe flow is that the flow disturbance becomes intermittently unstable in every pulsating cycle [2–5]. Notably, this intermittent flow behaviour has been found in the fully developed region [6–12]. According to Ohmi *et al*. [2] and Einav & Sokolov [12], the flow transition in the fully developed region of pulsating pipe flows can be categorized into three types: (i) laminar, in that the flow remains stable throughout the entire cycle; (ii) conditional or transitional turbulent, in that the flow becomes unstable within a certain phase region of one pulsating cycle and (iii) turbulent, in that the turbulent state prevails throughout the entire cycle. These flow types can be characterized by three parameters, namely, the mean and pulsating Reynolds numbers and the dimensionless pulsating frequency [2].

**Figure 1.**
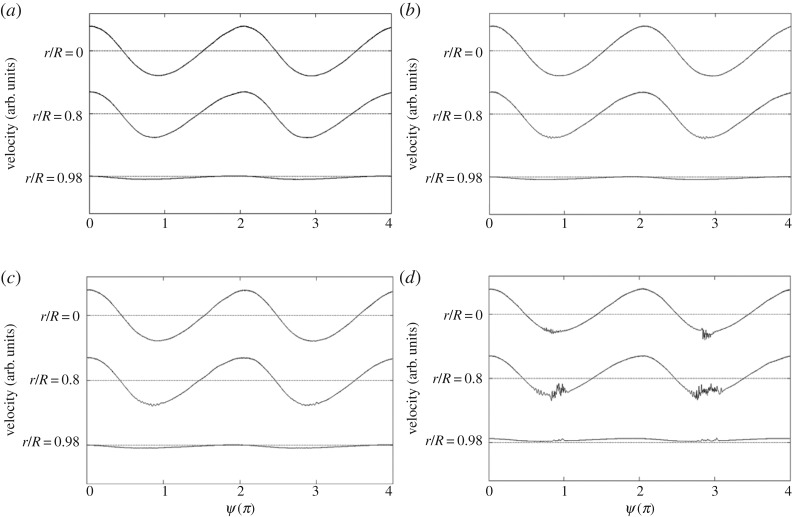
An illustration of the growth of flow instability in the pulsating pipe flow for (*Re_U_*, *Re_m_*, *α*) = (1.07 × 10^4^, 5.26 × 10^3^, 9.2) at *x*/*D* = 25 (*a*), 27 (*b*), 28 (*c*) and 29 (*d*). [15] The definitions of the experimental parameters of *Re_U_*, *Re_m_* and *α* are given in §2. Here *x* denotes the streamwise distance from the inlet of the straight pipe, *x* = 0. *D* and *R* denote the diameter and radius of the pipe, respectively. The phase-averaged velocity traces shown span two pulsating cycles, where *ψ* = 0 refers to the phase when flow pulsation reaches the maximum velocity at the centre of the pipe, *r* = 0.

Recently, Miau & Dai [13] and Miau & Jian [14] studied the flow instability that develops in the pipe's entrance region. The Reynolds numbers based on the time-mean velocity were of the order of 10^4^, which is substantially higher than the cases of flow transition taking place in the fully developed region, for which the Reynolds numbers were of the order of 10^3^ [7,8,12]. Figure 1 presents a case of the initial disturbance developed in the entrance region reported in a previous work [15]. The most upstream location where the unstable disturbance was found is at 27 pipe diameters downstream from the inlet. As seen, the initial disturbance appears as a packet of waves. Within a streamwise distance of two pipe diameters downstream, the disturbance grows and appears as turbulent fluctuations. Meanwhile, it is noted that the disturbance is diminished at certain times in later phases of the pulsating cycle period, signifying that the disturbance is damped and the flow regime returns to a stable state. This is referred to as a relaminarization process [2].

As noted in figure 1, the unstable disturbance initially appears as waves of small amplitude within a certain phase region of a pulsating cycle. This is typical of flow instability that develops in an unsteady mean flow. A similar appearance can be seen in an oscillating boundary layer [16].

In considering the physical mechanism associated with instability in a pulsating pipe flow, previous works frequently refer to Rayleigh's inflection-point theorem [17] as a theoretical ground for explanation since the velocity measurements show that the instantaneous velocity profiles might possess one or more inflection points. Sarpkaya [6] showed the existence of inflection points in the velocity profiles of the unsteady pipe flow obtained at different phases. Einav & Sokolov [12] analysed the velocity signals obtained in the fully developed region and postulated that the presence of an inflection point in the instantaneous velocity profile played a key role in the transition of the flow from a laminar to turbulent state. Nerem *et al*. [18] conducted hot-film velocity measurements in the thoracic aorta of dogs and noted that the fluctuations first appeared during the deceleration phase. Nerem *et al*. [18] commented that the fluctuations originated from flow instability could be due to the presence of an inflection point in the velocity profile. Moreover, it is worthwhile to mention the work of Gad-el-Hak *et al*. [19] on flow instability in a decelerating boundary layer. They commented that the inflection point in the decelerating boundary layer velocity profile promoted flow instability at a Reynolds number lower than the critical one corresponding to the steady boundary-layer flow. Both their experimental and theoretical results showed a consistent trend that the flow instability in the decelerating boundary layer developed as the inflection point lifted away from the wall.

Although the importance of the presence of an inflection point in unsteady mean flow to the occurrence of flow instability is well recognized, our understanding of its physical mechanism is far from complete. It is the purpose of this study to verify the role of inflection-point instability in the development of flow instability. To do so, the experimental data obtained were examined with a mixing-layer model, which is a physical case complied with the inflexion point instability theory.

## 2. Experimental method

**Figure 2.**
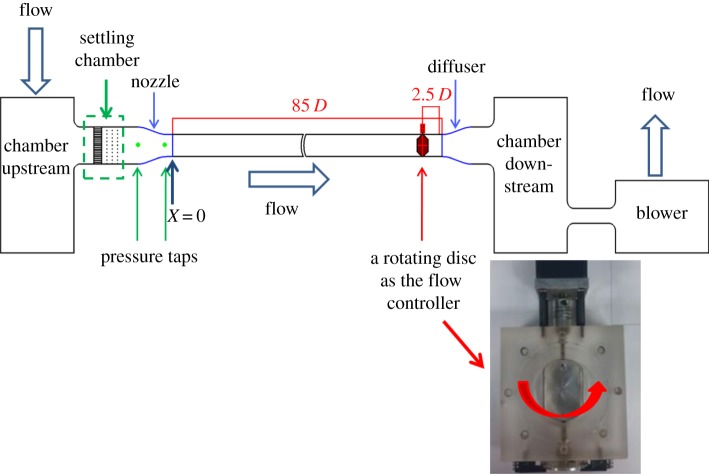
A schematic diagram of the pipe flow facility. (Online version in colour.)

Experiments were conducted in an open-circuit pipe flow system featuring a straight pipe section of 85 *D* in length, where *D* = 50 mm denotes the diameter of the pipe. See figure 2 for a schematic diagram of the pipe facility. A convergent section was situated immediately upstream of the pipe inlet to reduce the turbulence intensity of the flow at the inlet. The turbulence intensity measured at the core of the inlet is less than 1%. Two pressure taps at the inlet and outlet of the convergent section, respectively, were connected to a diaphragm-type pressure transducer for differential pressure measurement, by which the velocity was reduced for reference, denoted as *U_a_*. Under steady pipe flow conditions, *U_a_* could vary over a range of 3–50 m s^−1^.

Pulsating flow was produced by a rotating disc situated 82.5 *D* downstream from the inlet of the straight pipe section. As shown in figure 2, the disc was driven by a servo motor through a vertical shaft. A photo sensor was installed near the rotating disc to record the instant in time the disc passed over the sensor. The output signal was referenced for segmenting the simultaneously measured signal traces into individual sample records for ensemble-averaging. This process is known as phase-averaging. One revolution of the rotating disc actually generated two pulsating cycles of the pipe flow.

In this study, the pulsating flow condition can be described in terms of three independent parameters as follows. The mean Reynolds number *Re_U_* based on the time mean of *U_a_* and *D*. The pulsating Reynolds number *Re_m_* based on the amplitude of velocity modulation, *ΔU*, and *D*, where *ΔU* denotes the pulsating amplitude corresponding to the difference between the maximum and mean velocities measured at the core of the inlet of the pipe flow. The non-dimensional pulsating frequency, *α*, which is the Womersley number defined as *R*/*δ*, where *R* denotes the radius of the pipe, *R* = *D*/2, and *δ* = (*υ/ω*)^1/2^, characterizes the viscous diffusion thickness due to pulsation, where *υ* and *ω* denote the kinematic viscosity and the frequency of pulsation in radian/s, respectively. Here *δ* is also known as the thickness of the Stokes layer for an oscillatory flow [20,21].

Velocity measurements in the pipe flow were made with a boundary-layer type hot-wire probe. At a selected streamwise location, the hot-wire probe could be traversed from the centre of the pipe, *r*/*R* = 0, to a radial location very close to the wall, *r*/*R* = 0.98, where *r* denotes the radial distance from the centre of the pipe. The raw signals measured were sampled at a rate of 4 kHz over a time length of at least 30 revolutions of the rotating disc. Thus, referencing the optical sensor output, one could perform phase-averaging with regard to the velocity signals simultaneously measured.

## 3. Technique for resolving the disturbance frequency

The technique developed in previous studies [13,14] used to resolve the frequency of the intermittent flow disturbance seen in a pulsating cycle is briefly described below.

**Figure 3.**
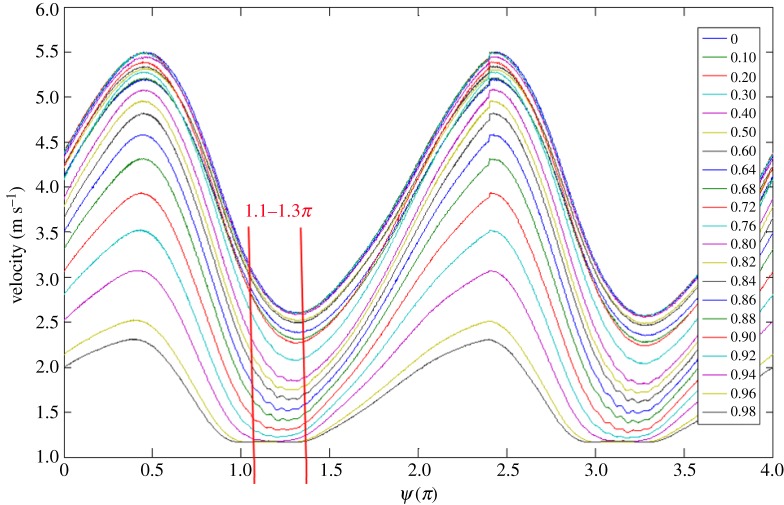
The phase-averaged pulsating velocity patterns measured at *r*/*R* = 0–0.98 for (*Re_U_*, *Re_m_*, *α*) = (1.1 × 10^4^, 4.3 × 10^3^, 12), *x*/*D* = 26. (Online version in colour.)

For illustration,the phase-averaged velocity traces obtained at *x*/*D* = 26 for *r*/*R* from 0 to 0.98, (*Re_U_*, *Re_m_*, *α*) = (1.1 × 10^4^, 4.3 × 10^3^, 12), are shown in figure 3, where *x* denotes the streamwise distance from the inlet of the pipe, called *x* = 0. As one can see, the flow disturbances appear most pronouncedly at *r*/*R* = 0.8–0.9 during the phase region of *ψ* = 1.1–1.3*π*. Moreover, it should be mentioned that the intermittent disturbance is fairly repeatable from one pulsating cycle to the other. Therefore, in figure 3 the wave form is preserved after the ensemble-averaging process. Meanwhile, the disturbance amplitude is so small that it can be treated as linear, mathematically speaking. The maximum amplitude of the disturbance in the individual pulsating cycle was confirmed within the order of 1% of the pulsating amplitude *ΔU*.

In figure 3, each of the ensemble-averaged velocity traces spans over two pulsating cycles, corresponding to one revolution of the rotating disc. In this analysis, only the ensemble-averaged trace in the first pulsating cycle is considered.

**Figure 4.**
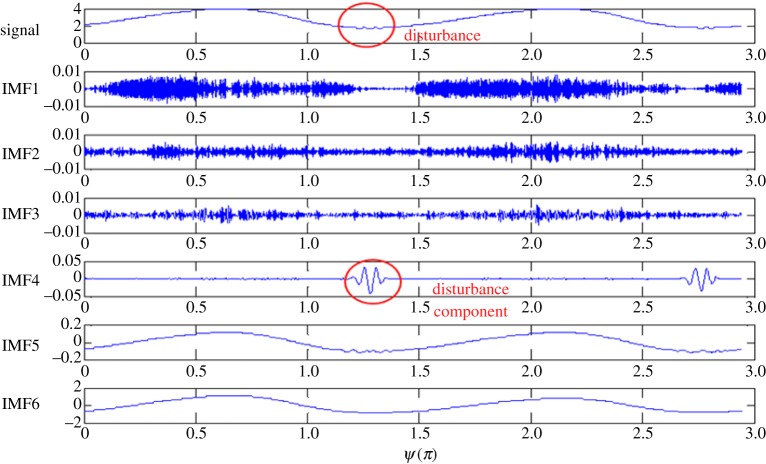
The EMD results of a raw hot-wire signal trace obtained at (*Re_U_*, *Re_m_*, *α*) = (1.1 × 10^4^, 4.3 × 10^3^, 12), *x*/*D* = 26, *r*/*R* = 0.88. IMF4 is identified as the disturbance component. (Online version in colour.)

The phase-averaged velocity trace at *r*/*R* = 0.88 in figure 3 is selected to illustrate how the embedded intermittent disturbance component was extracted for analysis. First of all, a procedure called empirical mode decomposition (EMD) [22] was used to decompose the trace into a set of mono-components, each of which is called an intrinsic mode function (IMF). A mono-component contains the fluctuations of the trace within a certain band of frequency. Ideally, the mono-components are mutually orthogonal [22]. The advantage of this procedure over the conventional band-pass filtering technique, as seen in figure 4, is that the disturbance component resembling a wave packet is well preserved in IMF 4. For more details concerning the EMD procedure, one may refer to Huang *et al*. [22].

**Figure 5.**
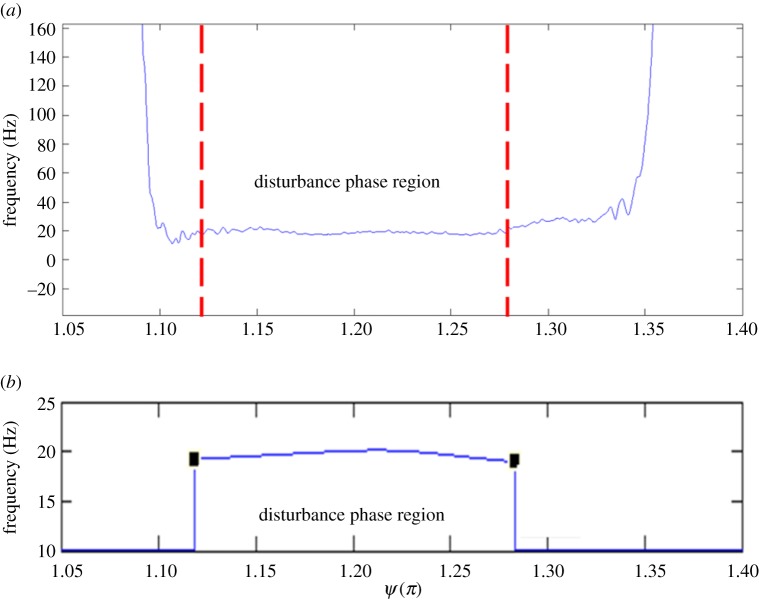
The disturbance frequency obtained by (*a*) Hilbert and (*b*) Wavelet transformations. The raw signal was obtained at (*Re_U_*, *Re_m_*, *α*) = (1.1 × 10^4^, 4.3 × 10^3^, 12), *x*/*D* = 26, *r*/*R* = 0.88. (Online version in colour.)

Further analysis on IMF 4 was carried out to find the characteristic frequency of the disturbance. The techniques of Hilbert [22] and Wavelet [23] transformations were considered, both of which are able to provide the instantaneous frequency for the disturbance component. As a result, figure 5*a*,*b* presents the instantaneous frequency of the disturbance resolved by the two techniques. Namely, by using the Hilbert transformation, the frequency is 19.1 ± 1.8 Hz; by using the Wavelet transformation, it is 19.6 ± 0.32 Hz. The uncertainty intervals mentioned refer to the 95% confidence interval [24] of the frequency values in the phase region where the disturbance is pronouncedly present. Since the uncertainty interval associated with the Wavelet transformation is significantly smaller than that which is associated with the Hilbert transformation, the Wavelet transformation technique was chosen for analysis. The larger scattering of the Hilbert transformation frequencies is partly due to the fact that the frequency resulted from the time differentiation of the instantaneous phase [22].

## 4. Considerations with Rayleigh inflection-point instability criterion

Reyleigh's inflection-point theorem [17] states that the presence of an inflection point in mean flow is necessary for the development of flow instability, assuming that the flow is inviscid. This theorem has been successfully applied to explain the linear instability growth in a mixing layer flow, for which an inflection point is identified in the mean velocity profile. As known, a mixing layer is formed at the interface of two parallel streams of different speeds. The inflection velocity profile actually results from the viscous action; however, the flow instability has been identified as an inviscid one.

**Figure 6.**
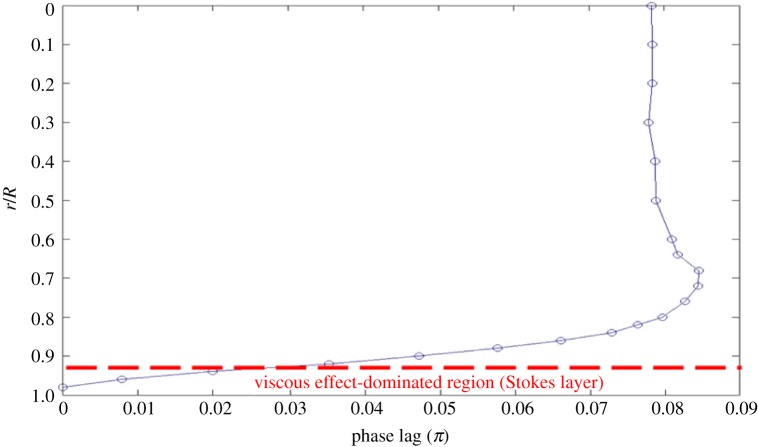
Phase lag distribution with respect to *r*/*R*, with the reference phase at *r*/*R* = 0.98. The experiment was made at (*Re_U_*, *Re_m_*, *α*) = (1.1 × 10^4^, 4.3 × 10^3^, 12), *x*/*D* = 26. (Online version in colour.)

To consider the formation of the inflection point in the present pulsating pipe flow, conceptually it can be explained as being due to the phase lag of the pulsating flow in the viscous boundary layer. Figure 6 shows a plot depicting the phase lag of the pulsating mean flow against *r*/*R* as reduced from the phase-averaged velocity data in figure 3. The phase lag was determined by referencing the phase of the unsteady mean flow at *r*/*R* = 0.98. Note that *r*/*R* = 0.98 is the measured location closest to the wall. This figure reveals that all the phase lag values are positive, inferring that flow pulsation away from the wall is always lagging behind flow pulsation near the wall. In addition, one can easily locate the outer edge of the viscous boundary layer. For instance, as shown in the figure, the outer edge is near *r*/*R* = 0.7. For *r*/*R* less than 0.7, the phase lag is approaching a constant value, indicating that potential flow prevails. On the other hand, one should be aware that a viscous effect-dominated layer caused by pulsation exists near the wall indicated in the figure. The thickness of this layer, which is called the Stokes layer, is estimated as *δ* = (*υ/ω*)^1/2^ that is *R*/*α*. Physically speaking, the phase lag in the Stokes layer is small, as the flow motion in this layer is dominated by the viscous force.

**Figure 7.**
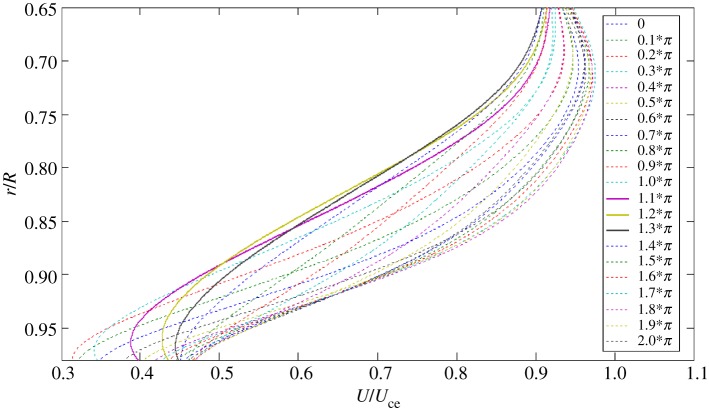
The fitting curves representing the normalized velocity profiles in the boundary layer over one pulsating cycle at the phases indicated. (Online version in colour.)

A direct consequence of pulsating flow phase lag in the boundary layer is that the instantaneous velocity profile is distorted and the inflection point may be formed. To demonstrate this possibility, the phase-averaged velocity profiles, *U*(*r*), corresponding to a number of phases *ψ* over one pulsating cycle, were reconstructed from the data in figure 3 for examination. The velocity profiles, each of which is actually a sixth-order polynomial curve fitting the phase-averaged data in figure 3, are shown in figure 7. In the figure, each velocity profile has been normalized by *U*_ce_, which denotes the phase-averaged velocity at *r*/*R* = 0 at the respective phase. However, the fitting curves are limited in the boundary layer region, because our main interest is the mean flow in the region. The correlation coefficient between each fitting curve and the corresponding phase-averaged velocity data was required to be at least 0.999 in value.

**Figure 8.**
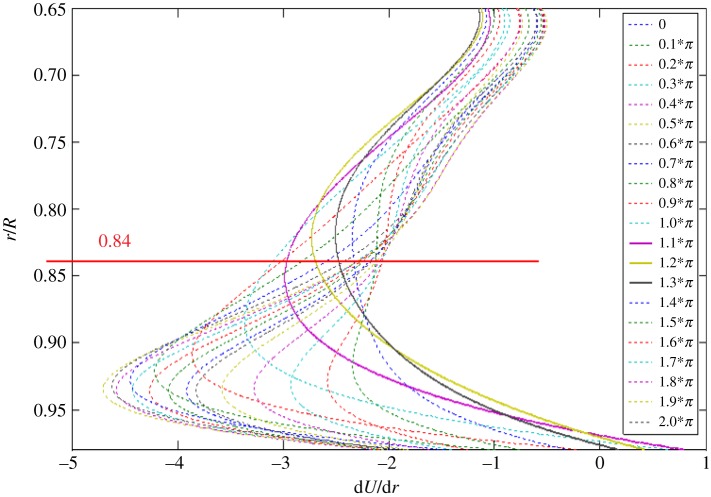
Plot of the first derivative of the fitting curves in figure 7. (Online version in colour.)

Figure 8 presents a plot of the first derivative of each fitting curve in figure 7. In the figure, d*U*/d*r* has been normalized by *U*_ce_/*R*. While each curve reveals the presence of an inflection point, where a local maximum value appears, those of *ψ* = 1.1–1.3*π* indicated by the solid curves unveil that the inflection points are situated further away from the wall. Referring to the phase-averaged velocity traces in figure 3, the phase *ψ* = 1.1*π* coincidentally is the instant when the disturbance initially appeared. At this phase, the inflection point is located near *r*/*R* = 0.84, whereas the edge of the Stokes layer is at *r*/*R* = 0.917, as indicated by a dashed line in figure 3.

**Figure 9.**
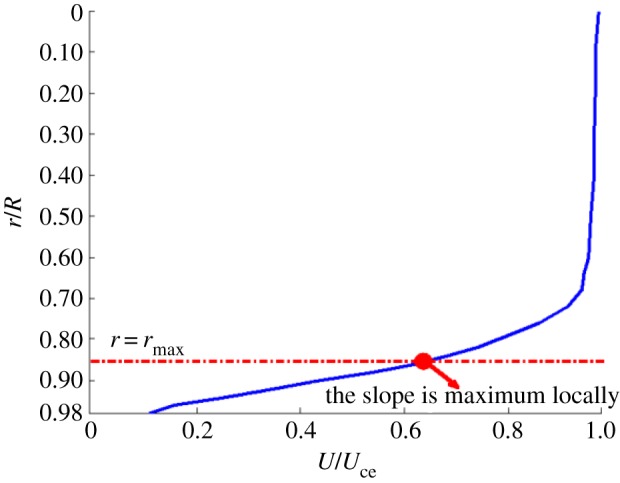
A physical model depicting the onset of flow instability in the entrance region. The solid curve represents an instantaneous boundary layer profile. (Online version in colour.)

To explain this observation, a physical model is proposed in figure 9. It is intended to show that the presence of an inflection point in the instantaneous velocity profile is due to the inconsistency of phase lag radially in the boundary layer. At the onset of the flow instability, an inflection point is formed in the mean flow at *r* = *r*_max_.

Subsequently, a discussion can be carried out with the mixing-layer model described below. Monkewitz & Huerre [25] conducted a numerical study on the linear instability of the hyperbolic tangent and Blasius mixing layers. They found that the non-dimensional frequency of the most unstable disturbance fell in the range of 0.2–0.25. Also noted is that this frequency range is rather insensitive to the basic flow profiles given. In Monkewitz & Huerre [25], the characteristic frequency of flow instability was normalized by the momentum thickness of the shear layer and the mean velocity of the two streams. Applying this mixing layer model to the present flow, as the case shown in figures 3–8, one postulates that the flow instability initiated at *ψ* = 1.1*π* was induced by a shear flow in the neighbourhood of the inflection point *r* = *r*_max_. As the characteristic frequency of the disturbance has been found to be 19.2 Hz, one can non-dimensionalize the frequency with the method described by Monkewitz & Huerre [25]. In figure 8, the velocity profile at *ψ* = 1.1*π* shows that an inflection point occurred near *r*/*R* = 0.84 with the d*U*/d*r* value about −3.0. Hence, the vorticity thickness equivalent to that of the mixing layer in the neighbourhood of the inflection point can be estimated as the absolute value of *R* divided by d*U*/d*r*. Further, following Monkewitz & Huerre [25], the momentum thickness can be estimated as a quarter of the vorticity thickness. Finally, by a rough estimation from taking the characteristic velocity of the equivalent mixing layer around the inflection point as 0.5 *U*_ce_, the disturbance frequency is non-dimensionalized to a value of 0.16, denoted as *Ω* herein. Qualitatively speaking, this value is close to the range of 0.2–0.25 mentioned for the mixing-layer model [25]. This agreement gives a strong support to our postulation that the development of the flow instability can be explained with the mixing-layer model.

It should be mentioned that the estimation above was made possible with a quasi-steady assumption that the flow instability was induced by the instantaneous velocity profile. This assumption can be justified by arguing that the time scale associated with mean flow pulsation is actually much longer than the time scale associated with the characteristic frequency of the unstable disturbance. For instance, in the case discussed in figures 3–8, the pulsating pipe flow was produced by a rotating disc at 17 r.p.m. Thus, one cycle of pulsation is equivalent to about 1.76 s. In comparison with the discovered disturbance frequency of 19.2 Hz, the characteristic time scale of unsteady mean flow is more than 30 times that of the disturbance.

## 5. Cases studied

The flow instability of concern varies strongly with time and space. Temporally, the flow instability grows and decays within a certain phase region of a pulsating cycle; spatially, as evidenced in figure 1, the flow instability is initiated at some streamwise location in the entrance region and leads to chaotic fluctuations within a few pipe diameters downstream. Therefore, to study the flow instability in the present flow, as the first step one needed to search for the streamwise location pertaining to the onset of flow instability. Namely, the discovered disturbance had to be so small in amplitude that it could be regarded as linear. Owing to this concern, in the following cases presented it was verified in advance that the maximum amplitude of the disturbance component at the selected streamwise location was comparable to 1% of the amplitude of mean flow pulsation at the core, *ΔU*.

### (a) Cases in agreement with the mixing-layer model

**Figure 10.**
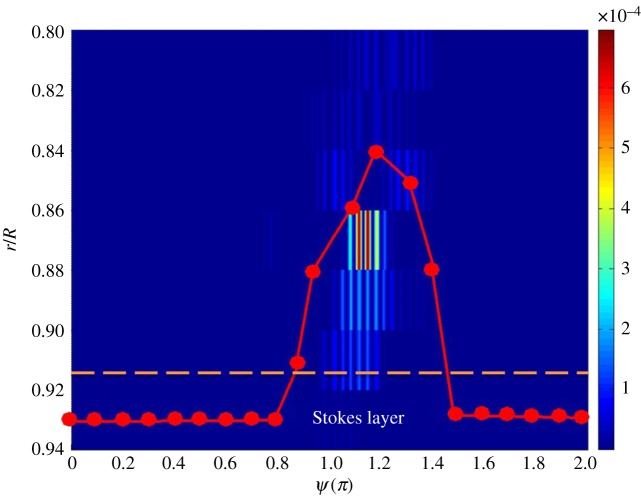
A comparison of the locus of the inflection points, *r* = *r*_max_(*ψ*), and the intensity of the disturbance with respect to *ψ*, for (*Re_U_*, *Re_m_*, *α*) = (1.1 × 10^4^, 4.3 × 10^3^, 12), *x*/*D* = 26. The dashed line indicates the edge of the Stokes layer, that is *r*/*R* = 0.917 in this case. This radial location is determined as *r*/*R* = 1 − 1/*α*. (Online version in colour.)

Figure 10 presents a plot that includes the locus of the inflection point and the intensity of the disturbance with respect to *ψ* based on the same set of the data shown in figure 3. Note that the intensity values of the disturbance shown in the figure are actually the square of the disturbance component of IMF 4 in figure 4, as reduced from the EMD procedure. As seen, the most pronounced intensity takes place at *r*/*R* = 0.86–0.88 and *ψ* = 1.1–1.2*π*. Coincidentally, the locus of the inflection point shows that at *ψ* = 1.2*π* the inflection point reaches the farthest point from the wall, that *r*_max_/*R* = 0.84. At later phases, the intensity of the disturbance decays as the inflection point moves towards the wall. As shown above, this case has been verified to be in agreement with the proposed mixing-layer model.

Table 1 lists five cases in this category. For these cases, the values of *Ω*, i.e. the non-dimensional frequencies of the onset of the disturbances, fall in the range of 0.13–0.2, which is comparable to the frequency range of the mixing-layer model mentioned [25]. Moreover, in the last column of the table, the non-dimensional quantity, *l*/*δ,* indicates how far the inflection point at the onset of the flow instability is situated from the wall, where *l* = *R −* *r*_max_. For these cases, the *l*/*δ* values are about two: the inflection point is situated two times the thickness of the Stokes layer from the wall. This information supports that the development of flow instability is attributed to the inviscid mechanism: namely, the mean flow in the neighbourhood of the inflection point modelled as a mixing-layer.

**Table 1.** Five cases in agreement with the mixing-layer model.

case	*X/D*	*α*	*Re_U_*	*Re_m_*	*f*_D_ (Hz)	*Ω*	*l*/*δ*
(i)	26	12	1.1 × 10^4^	4.3 × 10^3^	19.6	0.16	1.92
(ii)	26	12.6	1.2 × 10^4^	4.1 × 10^3^	20.1	0.13	2.01
(iii)	26	13	1.4 × 10^4^	4 × 10^3^	22.3	0.15	2.08
(iv)	31	10.9	1 × 10^4^	3.7 × 10^3^	15.3	0.2	1.74
(v)	31	12	9.9 × 10^3^	3.4 × 10^3^	17.3	0.19	1.92

### (b) Other cases

**Figure 11.**
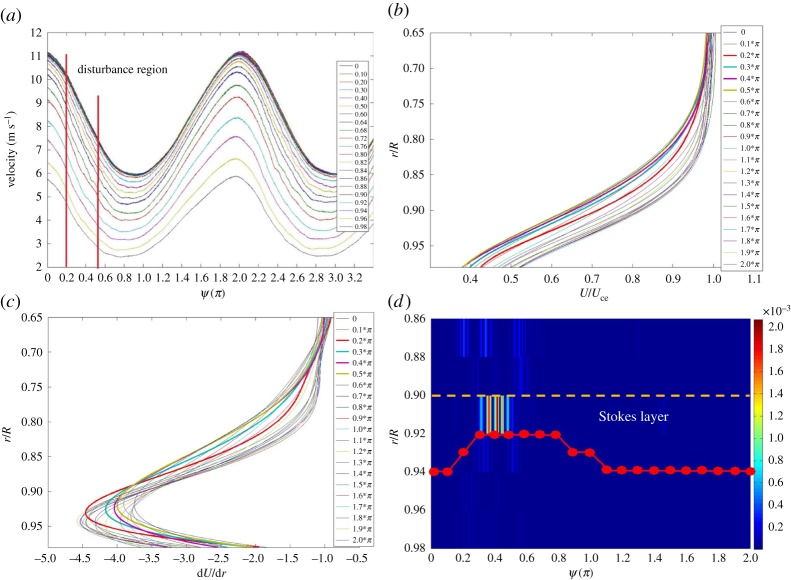
Results of (*Re_U_*, *Re_m_*, *α*) = (2.7 × 10^4^, 9 × 10^3^, 10), at *x*/*D* = 21. (*a*) Phase-averaged velocity patterns for *r*/*R* = 0–0.98; (*b*) the fitting curves representing the phase-averaged velocity profiles, *U*/*U*_ce_, in the boundary layer region; (*c*) the normalized d*U*/d*r* curves; (*d*) a comparison of the locus of the inflection points, *r *= *r*_max_(*ψ*), and the fluctuation intensity of the disturbance with respect to *ψ*. (Online version in colour.)

It should be pointed out that there are cases found in the experiment with disturbance characteristics that cannot be explained by the mixing-layer model proposed above. A case in this category is presented in figure 11 for illustration. For (*Re_U_*, *Re_m_*, *α*) = (2.7 × 10^4^, 9 × 10^3^, 10) and the velocity measurements made at *x*/*D* = 21 in the entrance region, figure 11*a* presents the phase-averaged velocity traces, from which it is seen that the disturbances developed when the flow was decelerating. In figure 11*b*, the normalized velocity profiles in the boundary layer corresponding to a number of phases over one period of pulsation look similar, unlike the case shown in figure 7. In figure 11*c*, one can realize that the inflection points of the unsteady mean flow profiles in figure 11*b* stay close to the wall even when the disturbance grows. Moreover, figure 11*d* reveals that a pronounced disturbance appears at the radial positions of *r*/*R* = 0.9–0.92, just inside the Stokes layer marked in the figure.

For this case, the disturbance component embedded in the ensemble-averaged velocity traces was extracted by the EMD procedures mentioned. Subsequently, the characteristic frequency of the flow disturbance was resolved by the Wavelet analysis. If the disturbance frequency non-dimensionalized in accordance with the mixing-layer model mentioned, the *Ω*-value would be 0.018. It is noted to be one order smaller than that predicted by the mixing layer model. Thus, this finding rather suggests that this case does not comply with the mixing layer model. In fact, based on the facts unveiled in figure 11 that the inflection point of the unsteady mean flow actually stay close to the Stokes layer over the entire pulsating cycle and the flow instability be developed within the Stokes layer, there is no justification of applying the inviscid argument in this case.

There are five cases of the category listed in table 2, whose *Ω*-values are consistently one order smaller than those of the cases in table 1. Also noted is that the corresponding *l*/*δ* values stay around one or even less. This indicates that these cases are not relevant to the inviscid instability model, contrary to the cases in table 1.

**Table 2.** Five cases not in agreement with the mixing-layer model.

case	*X/D*	*α*	*Re_U_*	*Re_m_*	*f*_D_ (Hz)	*Ω*	*l*/*δ*	$(2\pi f_{D}\nu )/U_{\text{ce}}^{2}$	$Re_{\delta *}$
(i)	21	10	2.7 × 10^4^	9 × 10^3^	8.94	0.018	0.8	1.25 × 10^−5^	1440
(ii)	21	12	2.5 × 10^4^	7.4 × 10^3^	6.44	0.021	0.79	1.05 × 10^−5^	1250
(iii)	26	12	2.6 × 10^4^	7.6 × 10^3^	6.63	0.02	0.99	1 × 10^−5^	1560
(iv)	31	10	2.7 × 10^4^	9.3 × 10^3^	6.4	0.011	0.96	8.95 × 10^−6^	1800
(v)	31	12	2.7 × 10^4^	8 × 10^3^	6.19	0.016	1.01	8.66 × 10^−6^	1575

## 6. Discussion

Further discussion is made below concerning the flow instabilities of the cases presented in tables 1 and 2. At first glance, one may quickly note that in table 1 the cases were performed at lower *Re_U_* in comparison with those in table 2. On the other hand, one may also note that the characteristic frequencies of the flow instability of the cases in table 1 are significantly higher those in table 2. Without knowing the physical mechanisms of the flow instabilities, this contradiction might create confusion.

As mentioned, the physical mechanism associated with the cases in table 1 is attributed to the inflection-point of the mean flow that is inviscid in nature. In fact, during the experiment these cases were produced with the same rotating disc whose area blockage was 85% of the cross-sectional area of the pipe. By varying the Reynolds number *Re_U_*, one can see that the streamwise location where the initial disturbance was observed varied accordingly. Namely, the lower the Reynolds number *Re_U_*, the further downstream the initial disturbance appears, as seen in cases (iv) and (v). On the other hand, it should be mentioned that the flow instability was also dependent upon the pulsation amplitude in terms of the Reynolds number *Re_m_*. In the experiment, the Reynolds number *Re_m_* could be lowered by employing a rotating disc of smaller size: for instance, an area blockage ratio of 70%. However, by doing so, one found no flow instability in the entrance region at the same *Re_U_* and *α*.

The cases shown in table 2 were produced by the rotating disc with a blockage ratio of 70% at *Re_U_* significantly higher than the *Re_U_* in table 1. During the experiment, these cases were viewed by increasing the pipe flow velocity in a stepwise fashion until the flow instability was discerned in the entrance region. As seen, the Reynolds numbers *Re_U_* of the cases in table 2 are more than two times those shown in table 1, while the *α* values of these cases are comparable.

**Figure 12.**
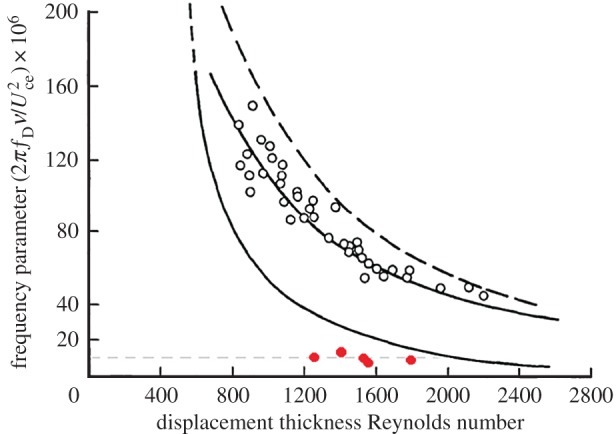
The marginal stability curve of a Blasius boundary layer [16]. The open symbols correspond to the oscillatory boundary layer [16]; the solid symbols correspond to the cases in table 2. (Online version in colour.)

The flow instability associated with the cases of table 2 can be attributed to the viscous mechanism, as explained below. Literally speaking, the flow instability seen is in the unsteady boundary layer developed in the entrance region. Referring to Obremski & Fejer [16] and Gad-el-Hak *et al*. [19], one may consider their viewpoint and adopt the stability theory of a steady laminar boundary layer to examine the present flow instability. Following Gad-el-Hak *et al*. [19], a quasi-steady assumption is also made here that the flow instability is induced by the instantaneous velocity profile. With the characteristic frequencies of the initial disturbances given in table 2, one can non-dimensionalize the frequency, *f*_D_, as $(2\pi f_{D}\nu )\text{/}U_{\text{ce}}^{2}$ [16], where *ν* denotes the kinematic viscosity. The non-dimensional values are found to be of the order of 1 × 10^−6^. Meanwhile, the Reynolds numbers based on the displacement thickness of the boundary layers at the streamwise locations measured are estimated to be in the range of 1500–2000. This estimation was enabled by the boundary layer thickness obtained from the phase lag plot of pulsating flow against *ψ*. Subsequently, the displacement thickness was taken as one-third the boundary layer thickness like the Blasius boundary layer profile [26]. In table 2, these values from the five cases are listed for reference. These cases are close to the marginal stability curve of the Blasius boundary layer profile [16], which is shown in figure 12. Moreover, according to Gad-el-Hak *et al*. [19], the region defined by the marginal stability curve corresponding to a decelerating boundary layer could be enlarged, in comparison with that of the Blasius boundary layer. Therefore, the cases in table 2 could be even closer to the marginal stability of a boundary layer in deceleration. Nevertheless, more quantitative investigations on the marginal instability of the present flow would be necessary in the future.

**Figure 13.**
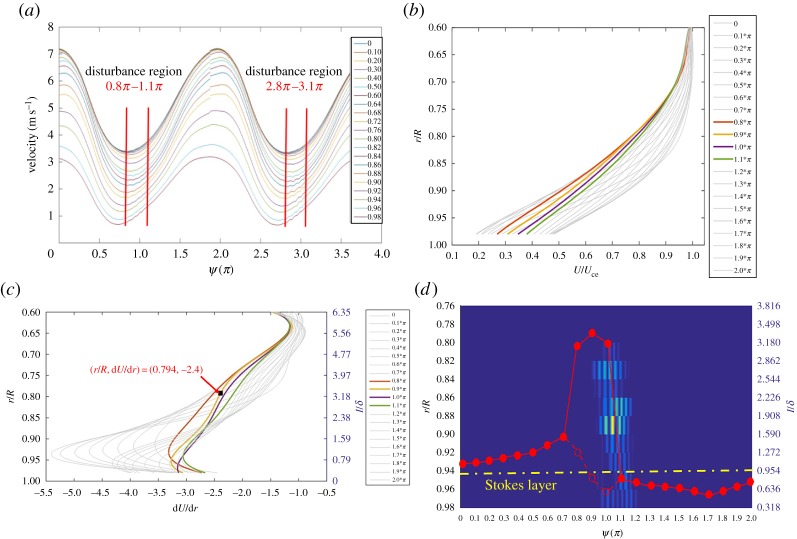
Results of (*Re_U_*, *Re_m_*, *α*) = (1.7 × 10^4^, 6.4 × 10^3^, 15.9), at *x*/*D* = 21. (*a*) Phase-averaged velocity patterns for *r*/*R* = 0–0.98; (*b*) the fitting curves representing the phase-averaged velocity profiles, *U*/*U*_ce_, in the boundary layer region; (*c*) the normalized d*U*/d*r* curves; (*d*) a comparison of the locus of the inflection points, *r* = *r*_max_(*ψ*), and the fluctuation intensity of the disturbance with respect to *ψ*. (Online version in colour.)

A case of inviscid flow instability is presented in figure 13 for discussion. This case was made at (*Re_U_*, *Re_m_*, *α*) = (1.7 × 10^4^, 6.4 × 10^3^, 15.9). The initial flow instability was observed at *x*/*D *= 21. In figure 13*a*, the ensemble-averaged velocity traces obtained at *x*/*D *= 21 over two pulsating cycles indicate that the intermittent disturbance appears at the phase when the unsteady mean flow near the wall is accelerating. With the fitting curves in figure 13*b* representing the velocity profiles in the unsteady boundary layer, the first derivative curves are presented in figure 13*c*. In the plot, the curves thicker in width correspond to the phases when the flow disturbances are clearly visible. The curve of *ψ* = 0.9*π* is chosen in particular for examination. As noted, there are two inflection points in this curve; one is immersed in the Stokes layer and the other is situated about three times the thickness of the Stokes layer from the wall. The characteristic frequency of the disturbance resolved is 28.5 Hz. Non-dimensionalizing the frequency in accordance with the mixing-layer model with respect to the inflection point situated outside the Stokes layer gives the *Ω*-value 0.178. This value is noted to be comparable to those shown in table 1. Furthermore, in figure 13*d*, the locus of the inflection points deviating away from the Stokes layer, which is seen in the solid curve, coincides with the development of the intermittent disturbance. Consequently, it gives evidence that the disturbance grows outside the Stokes layer while the inflection point is lifting away from the wall. Based on the observations above, one can state that the flow instability is governed by the inviscid mechanism, although the unsteady mean flow has one more inflection point existing in the Stokes layer that is marked by the dashed symbols.

This case provides an example showing that an unsteady mean flow could have multiple inflection points while the inviscid flow instability dominates. In figure 13*c*, it is seen that although the inflection point away from the Stokes layer is only vaguely identified, it is so effective that it governs the development of flow instability. Since the Reynolds number *Re_U_* of this case is between those of tables 1 and 2, one would expect that if *Re_U_* were somewhat higher, the flow instability could have a different result. In general, the flow instability seen at lower *Re_U_* is triggered by the inviscid mechanism, and at higher *Re_U_* it is overtaken by the viscous mechanism. Conceivably, a competition between the inviscid and viscous instability mechanisms would take place if *Re_U_* falling in the intermediate range.

## 7. Concluding remarks

This study confirms a flow regime in pulsating pipe flow that the inviscid flow instability prevails in the entrance region. In comparison with the cases governed by the viscous mechanism, the inviscid flow instability regime is found at lower *Re_U_* and higher *Re_m_*, such that the inflection point situated outside of the Stokes layer plays an effective role.

Comparing the present work with other research concerned with the flow transition in the fully developed pipe flow region, one can see a major difference in that the Reynolds number *Re_U_* considered in the present flow is of the order of 10^4^, which is significantly higher than those of studies in the fully developed region. For instance, the experiments of Shemer *et al*. [7] and Settler & Hussian [8] were made with the Reynolds numbers in the range of 2000–4000. In the present flow, higher *Re_U_* caused the flow instability to take place in the entrance region.

While the flow instability developed in the entrance region of the pipe flow is initiated in the viscous boundary layer, the results of this study clarify that the mechanism is not necessarily a viscous one. This should be differentiated from the previous studies concerned with unsteady boundary layers [16,19]. In fact, two types of flow instabilities were discussed in this work. Generally speaking, at lower *Re_U_* the flow instability is triggered by the inviscid mechanism; at higher *Re_U_* it is overtaken by the viscous mechanism.

Moreover, the case in figure 13 reveals a situation where the flow instability is actually developed as the pulsating flow is accelerating. This would be highly unlikely from the perspective of the viscous instability mechanism. This study provides the experimental data with analysis to give evidence that this flow phenomenon is due to the inviscid mechanism: namely, the inflexion-point instability.

Additional remarks on the studies concerning the flow instability of an oscillatory pipe flow are worthwhile to be made here. The oscillatory pipe flow is referred to an unsteady pipe flow with zero mean velocity. Hino *et al*. [27] conducted an experimental work to study the phenomenon of laminar–turbulent transition in an oscillatory pipe flow. They identified three types of turbulent flow regimes, namely, weakly turbulent, conditionally turbulent and fully turbulent, characterized by the Reynolds number *R_δ_*, based on the oscillatory velocity amplitude and the thickness of the Stokes layer, and the Stokes parameter equivalent to the Womersley number defined in this work. Eckmann & Grotberg [28] conducted the velocity measurements in an oscillatory pipe flow over a range of the Reynolds number and Womersley number to study the flow transition to turbulence. As they found, the transition was detected during the decelerating phase of flow, and the turbulence was confined in an annular region a few times the Stokes-layer thickness near the wall. Later, Blennerhassett & Bassom [21] conducted a numerical study on flow instability of oscillatory pipe flows. They found for a case of the pipe diameter being about ten times the Stokes-layer thickness that the velocity perturbation corresponding to the least damped mode bears the resemblance to those reported by Hino *et al.* [27] and Eckmann & Grotberg [28]. In these studies, discussions made were mainly concerned with the viscous effect due to the oscillatory flows. Whether or not the inviscid mechanism reported in this study would play a role in the oscillatory pipe flows is of interest to be considered in the future.

## Data accessibility

The data reported in this paper are available upon request.
